# Repeat endoscopic submucosal dissection of esophageal squamous cell carcinoma near a previous scar by water pressure method

**DOI:** 10.1055/a-2643-8690

**Published:** 2025-08-08

**Authors:** Kazuo Shiotsuki, Kohei Takizawa, Shuntaro Ishikawa, Mitsuhiro Furuta, Nozomu Machida, Takashi Ogata, Shin Maeda

**Affiliations:** 1 91321 Department of Gastroenterology, Kanagawa Cancer Center, Kanagawa, Japan; 2 91321 Department of Gastrointestinal Surgery, Kanagawa Cancer Center, Kanagawa, Japan; 3 Department of Gastroenterology, Yokohama City University Graduate School of Medicine, Yokohama, Japan

Endoscopic submucosal dissection (ESD) is a standard treatment for superficial esophageal squamous cell carcinoma (ESCC). Metachronous ESCC sometimes occurs close to the previous ESD scars, and a repeat ESD becomes difficult due to the high risk of perforation 1 . A previous ESD can cause severe fibrosis in the submucosal layer, making it difficult to identify the appropriate dissection layer and successfully perform a repeat ESD. Recently, a water pressure method (WPM), which facilitates easy access to the mucosal flap, was developed 2 3 , and some reports have shown that it can help with ESD in patients with severe fibrosis 4 5 . Herein, we present a case in which WPM helped perform an en bloc resection of ESCC, located at a site overlapping a previous ESD scar, without perforation ( Video 1 ).

Successful repeat endoscopic submucosal dissection of an esophageal squamous cell carcinoma close to the previous ESD scar using the water pressure method.Video 1

An 82-year-old man presented with a history of ESD of ESCC in the upper thoracic esophagus. Esophagogastroduodenoscopy showed metachronous ESCC located overlapping the scar from the previous ESD, and iodine staining revealed that the tumor had spread to approximately 7/8th of the circumference of the esophageal lumen ( Fig. 1 **a** ).

**Fig. 1:**
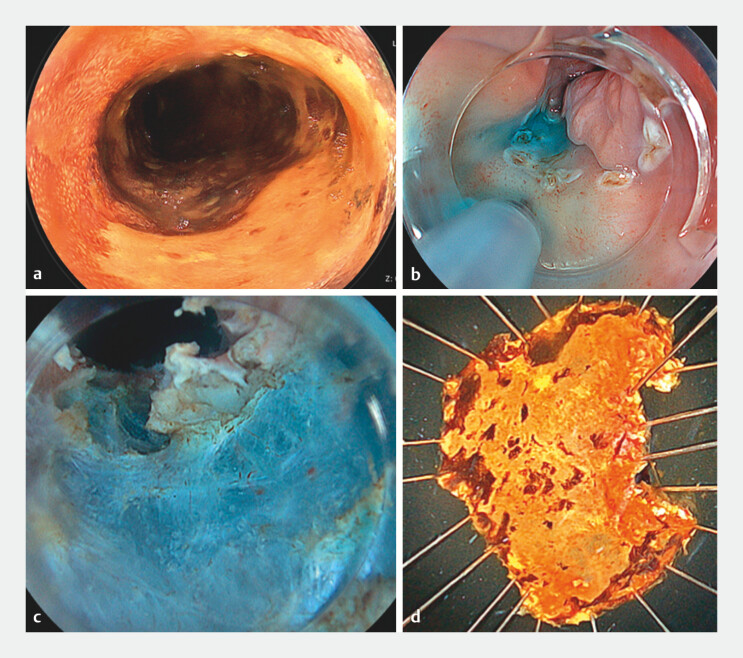
**a** Using esophagogastroduodenoscopy, iodine staining revealed that the tumor spread to approximately 7/8th of the circumference of the esophageal lumen. **b** After injecting hyaluronic acid, a non-lifting sign was observed, owing to severe fibrosis. **c** The water pressure method improved the visibility of the appropriate dissection layer with buoyancy and natural magnification effect. **d** The water pressure method helped achieve en bloc resection without perforation.

ESD was performed under general anesthesia. An ultrathin therapeutic endoscope (EG-840TP; FUJIFILM, Japan) was used since a standard endoscope could not pass through owing to the stenosis. In addition, an energy device (Dual knife J, IT-knife nano; Olympus) and an electrosurgical unit (VIO3; ERBE, Tubingen) were used. After injecting hyaluronic acid at the oral side, close to the previous ESD scar, a non-lifting sign was observed due to severe fibrosis ( Fig. 1 **b** ). The lumen was filled with saline, water pressure was applied to the incision line, and due to buoyancy, a slight flap was formed that helped identify the appropriate dissection layer ( Fig. 1 **c** ). The WPM method, therefore, guided the dissection of severe fibrosis, enabling en bloc resection without perforation ( Fig. 1 **d** ).

Endoscopy_UCTN_Code_TTT_1AO_2AG_3AD

## Notes

Endoscopy E-Videos https://eref.thieme.de/e-videos
*E-Videos* is an open access online section of the journal *Endoscopy* , reporting on interesting cases and new techniques in gastroenterological endoscopy. All papers include a high-quality video and are published with a Creative Commons CC-BY license. Endoscopy E-Videos qualify for HINARI discounts and waivers and eligibility is automatically checked during the submission process. We grant 100% waivers to articles whose corresponding authors are based in Group A countries and 50% waivers to those who are based in Group B countries as classified by Research4Life (see: https://www.research4life.org/access/eligibility/ ). This section has its own submission website at https://mc.manuscriptcentral.com/e-videos .
